# Case report: Myocardial perfusion gated-SPECT in pulmonary artery hypertension—the Movahed's sign

**DOI:** 10.3389/fcvm.2023.1168360

**Published:** 2023-07-27

**Authors:** Kevin Hamzaraj, Silvia Angjeliu, Paul Knopf, Michael Stadler, Kamil Zbucki, Lisbona Kastrati, Senta Graf, Mariann Gyöngyösi, Marcus Hacker, Raffaella Calabretta

**Affiliations:** ^1^ Division of Cardiology, Department of Internal Medicine II, Medical University of Vienna, Vienna, Austria; ^2^ Division of Nuclear Medicine, Department of Biomedical Imaging and Image-Guided Therapy, Medical University of Vienna, Vienna, Austria

**Keywords:** myocardial gated-SPECT, pulmonary artery hypertension, right ventricle, case report, myocardial perfusion

## Abstract

Primary pulmonary artery hypertension (PAH) is a clinical diagnosis that requires the exclusion of other underlying causes of pulmonary hypertension (PH). Increased pulmonary artery (PA) pressure and subsequent right ventricular (RV) pressure overload often result in a flattening of the curved interventricular septum, leading to a D-shaped left ventricle (LV), as observed in echocardiographic short-axis views. A similar finding may be also observed on myocardial perfusion SPECT images, the so-called Movahed's sign. We present a clinical case of a female patient with PAH and progression of exertional dyspnea that underwent myocardial perfusion SPECT to investigate LV myocardial ischemia. The SPECT images revealed enhanced tracer uptake in the dilated right ventricle. Additionally, we observed a D-shaped LV or Movahed's sign, which may serve as a potential marker of RV pressure overload, along with a small stress-induced perfusion defect on the LV septal wall. Our findings highlight the importance of considering the presence of a D-shaped LV and signs of RV pressure overload, as they can alter the interpretation of LV perfusion deficits on SPECT images. This case report aims to emphasize the complex nature of right heart abnormalities in pathologies such as PAH and the consideration of the RV implications in myocardial SPECT images—which typically focus solely on the LV.

## Introduction

Primary pulmonary artery hypertension (PAH) is characterized by abnormal echocardiographic findings of precapillary origin. Right ventricular (RV) overload is a common finding in patients with PAH and is primarily investigated using echocardiography, cardiac magnetic resonance imaging, and right heart catheterization (1). In general, patients with primary PAH may report non-specific symptoms including shortness of breath, chest pain, or chest discomfort, resembling those of coronary artery disease (CAD) (2). Therefore, in diagnosing PAH, it is essential to rule out left ventricular (LV) myocardial ischemia due to CAD. In cases where CAD is suspected or known, electrocardiogram (ECG)-gated single-photon emission computed tomography (SPECT) is a suitable non-invasive option for assessing LV myocardial perfusion and function (1–4). Generally, myocardial perfusion SPECT images typically lack radioactive tracer uptake in the healthy RV, owing to its lower vascularization and thinner wall, limiting the analysis of RV perfusion and function (2, 5). PAH can exert morphological effects on both cardiac ventricles, leading to RV dilation and hypertrophy, resulting in interventricular septum flattening towards the LV, commonly manifested as a D-shaped LV (6). Interestingly, a similar D-shaped LV, indicating RV overload, can also be observed on myocardial SPECT imaging and is known as the Movahed's sign (7).

We report a case of a female patient with known PAH who underwent myocardial gated- SPECT to investigate a probable presence of CAD, due to the progression of exertional dyspnea.

## Patient information

A 62-year-old female patient presented to the Outpatient Clinic of the Department of Cardiology at the Medical University of Vienna for her periodic 6-month routine visit to review her cardiovascular (CV) medication. Her past medical history included systemic hypertension, hypercholesterinemia, and a previously diagnosed precapillary PAH dating back to 2013. Since 2014, the patient has been receiving Treprostinil pump therapy with a concentration of 10 mg/ml starting with 140 ng/kg/min. Over the years, routine visits led to dose adjustments, eventually resulting in a dose up-titration of up to 268 ng/kg/min in 2019. Since 2021, a progression in pulmonary artery pressures as a non-responder to vasoreactivity testing could be observed, despite the absence of dyspnea in the patient's daily life.

## Clinical findings and diagnostic assessment

**Figure 1:**
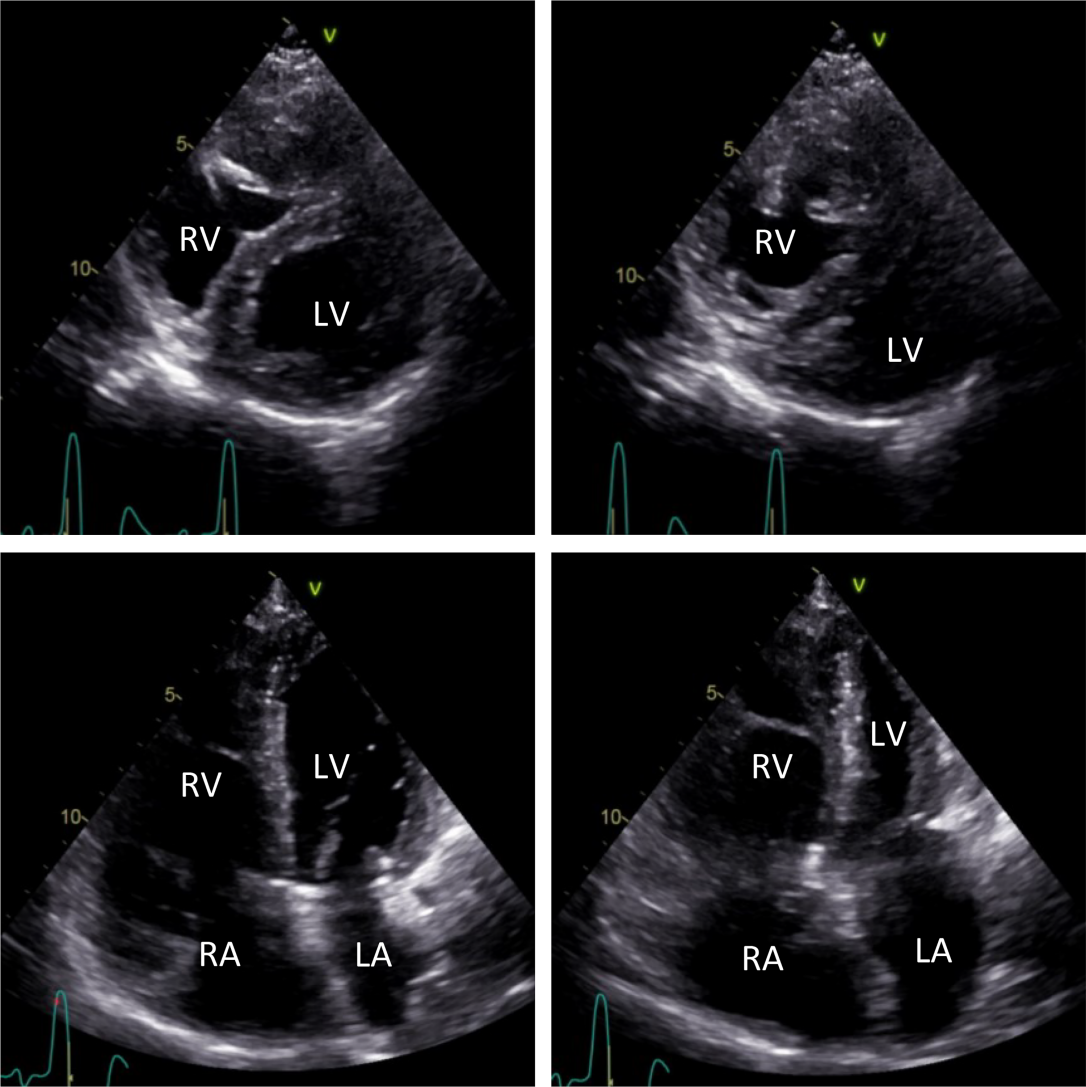
Transthoracic echocardiographic short-axis images (top) and long-axis four-chamber images (bottom). Echocardiographic short-axis views show a D-shaped, mildly hypertrophic LV during diastole (left) and systole (right). Mild dilation of the RA and RV can be observed in echocardiographic long-axis four-chamber views during diastole (left) and systole (right). LV, left ventricle; LA, left atrium; RA, right atrium; RV, right ventricle.

Recently, the patient presented to the Outpatient Clinic of the Department of Cardiology with exertional dyspnea (NYHA III) persisting for the previous 3 months. NT-proBNP value was 240 pg/ml. A transthoracic echocardiogram was performed and revealed mild LV hypertrophy with normal systolic LV function, a slight LV diastolic function reduction, and abnormal septal motion. D-shaped LV was demonstrated on echocardiographic short-axis views during systole (Figure 1).

Furthermore, the patient showed mild dilation of the right atrium (RA) and the RV (Figure 1), along with mild tricuspid regurgitation and a systolic PA pressure of 83 mmHg. The aneurysmatic dilated PA had a diameter of 52 mm. No further valvular abnormalities were detected during the echocardiographic examination.

Considering the recent symptom progression and absence of recent angiographic information on coronary arteries, the patient was referred to the Division of Nuclear Medicine at the Medical University of Vienna to rule out LV myocardial ischemia associated with CAD.

The patient underwent myocardial perfusion ECG-gated SPECT with [^99m^Tc]Tc-Tetrofosmin, after pharmacological stress with Regadenoson® and at rest, conducted according to the 1-day protocol of our institution and using the D-SPECT®Cardio camera (Spectrum Dynamics Medical) in a sitting position. The baseline ECG findings, before the stress test began, were sinus rhythm, 89/min, P-mitrale, bifascicular block (left anterior hemiblock and right bundle branch block—QRS 148 ms), ST-segments not significantly pathological, terminal negative T-waves in aVL, V1–V4. During the stress test, as well as in the recovery phase, no specific ECG modifications and no specific symptoms were recorded. The patient's blood pressure was 95/60 mmHg at rest, 120/70 mmHg during hyperemia, and 110/65 mmHg during the recovery phase.

Rest and stress SPECT studies were reconstructed using state-of-the-art imaging software protocols provided by Spectrum Dynamics Medical Processing Station 4.2.3 and analyzed on the Hermes Medical Solutions (Stockholm, Sweden) platform. Then, the reconstructed images were realigned along the standard cardiac planes (short axis, vertical long axis, and horizontal long axis). The [^99m^Tc]Tc-Tetrofosmin LV tracer uptake, interpreted as LV myocardial perfusion, was qualitatively and semi-quantitatively assessed using a 5-point scale on the 17-segments scheme, obtaining the summed rest (SRS), summed stress (SSS), and summed difference scores (SDS). The measurement of LV function as LV ejection fraction (LVEF), LV end-diastolic, and end-systolic (EDV, ESV) volumes was additionally performed by automated and validated methods using state-of-the-art imaging software QGS/QPS (Cedars-Sinai Medical Centre, Los Angeles, California).

**Figure 2:**
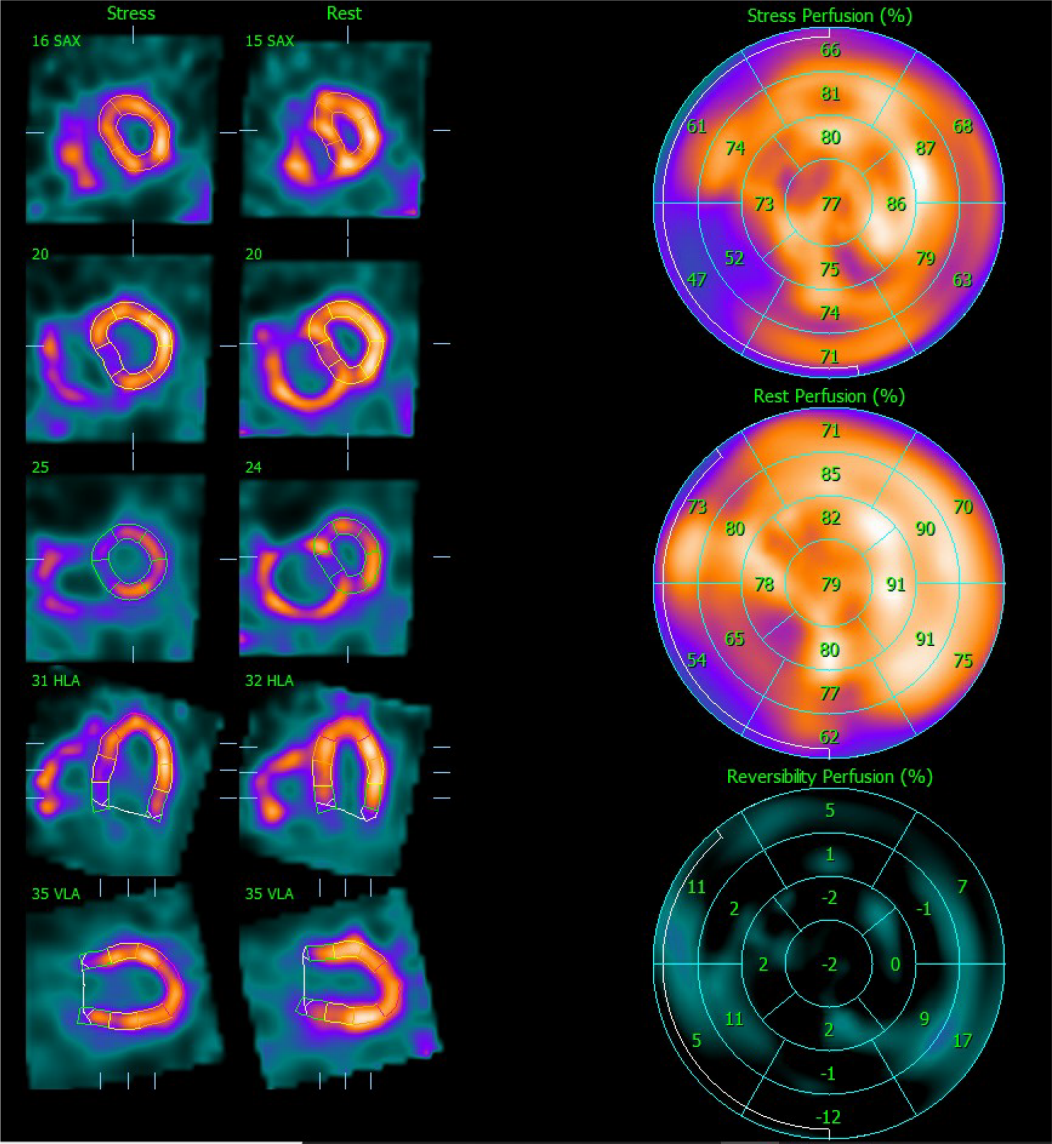
Myocardial perfusion SPECT images after stress and at rest. On the left side, the representation of the LV after stress and at rest is realigned along the three standard cardiac planes (short axis, vertical long axis, and horizontal long axis). On the right side, polar maps (stress, rest, and reversibility) illustrating the [^99m^Tc]Tc-Tetrofosmin uptake interpreted as LV perfusion assessed semi-quantitatively using the percent of the uptake on the 17-segment scheme. SPECT, single photon emission computer tomography; LV, left ventricle.

**Figure 3:**
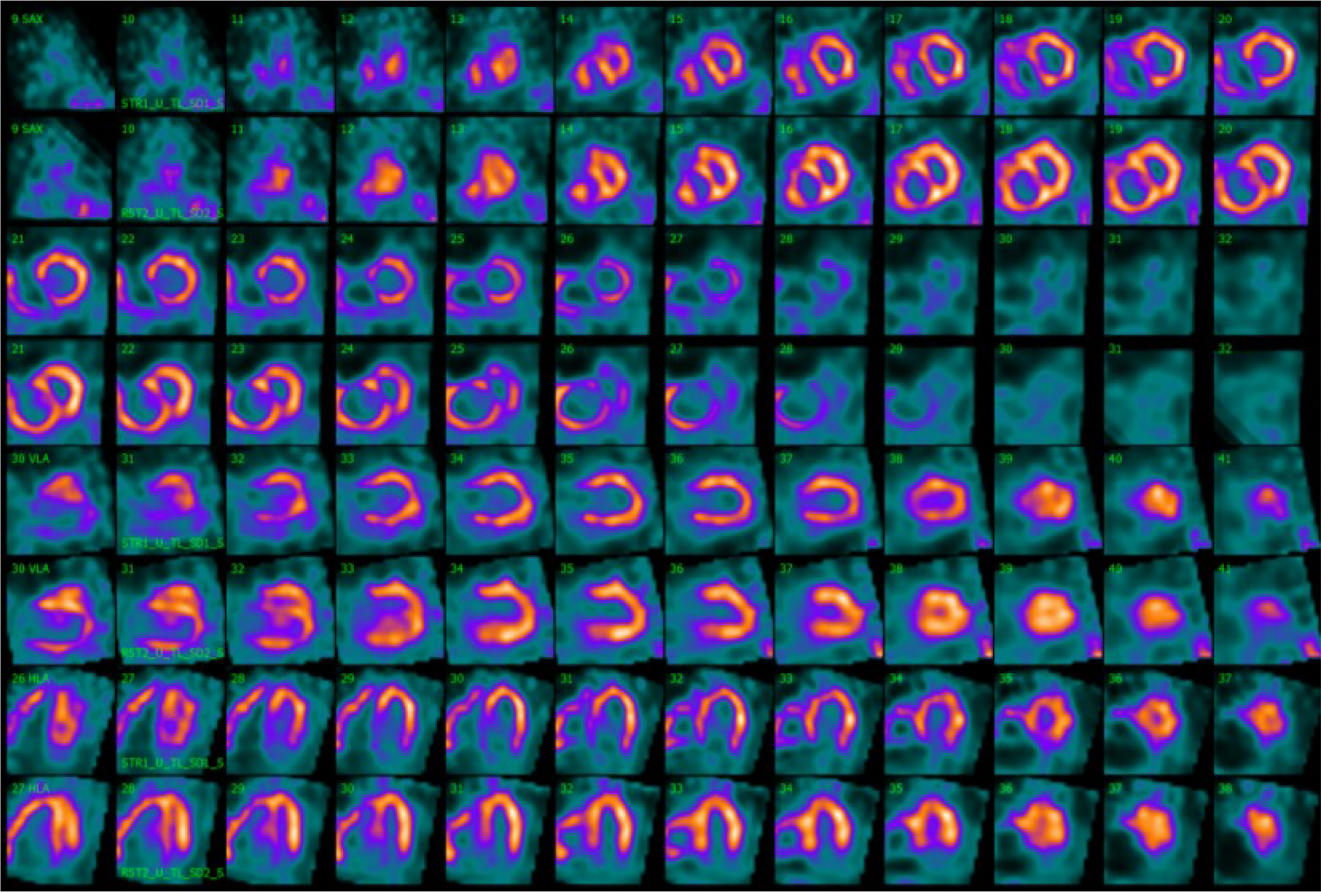
Movahed's sign on myocardial perfusion SPECT is observed on the splash image view. Myocardial perfusion SPECT images after stress (even rows) and at rest (odd rows) realigned along the three standard cardiac planes (short axis, vertical long axis, and horizontal long axis). Representation of a severely dilated RV with massive wall tracer uptake and a flattening of the interventricular septum resulting in a D-shaped LV, also called Movahed's sign. SPECT, single photon emission computer tomography; RV, right ventricle; LV, left ventricle.

Stress LV perfusion images showed a moderate perfusion defect in the mid-basal inferoseptal wall and a small perfusion defect in the basal segment of the inferolateral wall (SSS 5). At rest, we could observe a recovery of both perfusion defects as well as diffuse improvement of the LV perfusion (SRS 0), with an SDS of 5 (Figure 2). Gated acquisitions demonstrated normal LV function (LVEF > 60%), EDV, and ESV in both studies. No significant abnormalities were noted in LV wall motion and thickening analysis post-stress or at rest. Notably, the SPECT images displayed a non-typical finding—a heterogeneous and significantly enhanced tracer uptake in the RV wall, indicating increased vascularization and hypertrophy of the severely dilated RV. Additionally, the flattening of the interventricular septum resulting in a D-shaped LV suggests the presence of RV overload (Figure 3).

**Figure 4:**
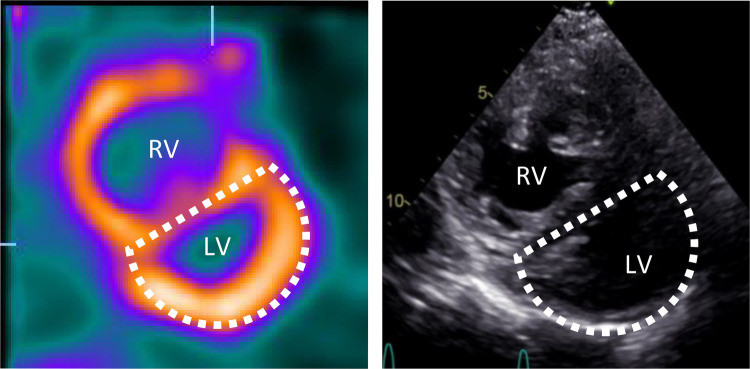
Movahed's sign on myocardial perfusion SPECT and D-shaped left ventricle on echocardiography. Short axis myocardial perfusion SPECT images at rest (left) and short axis view on transthoracic echocardiography (right) show the D-shaped left ventricle. SPECT, single photon emission computer tomography; RV, right ventricle; LV, left ventricle.

The D-shaped LV observed on myocardial perfusion SPECT was consistent with the analogous sign observed on echocardiographic short-axis views during systole (Figure 4). The perfusion defects were interpreted in relation to the RV pressure overload, with a low likelihood of coronary artery stenosis. Consequently, no additional diagnostic coronary angiography was performed. Furthermore, despite the progression of dyspnea, it was unlikely to be attributed to the small ischemic area without a clearly identifiable responsible vessel. Nevertheless, the option of a coronary CT or calcium scoring was discussed with the patient as a potential further diagnostic measure to fully rule out a CAD.

## Discussion

Primary PAH is a precapillary PH subgroup characterized by increased mean PA pressure (PAP) exceeding 25 mmHg (1). The exact pressure can only be measured invasively through right heart catheterization. While non-invasive assessment of pressures using echocardiography may appear sufficient to establish diagnostic criteria, other echocardiographic measurements are necessary to evaluate the probability of PH. Within this context, RV pressure is elevated due to increased PAP (8). Echocardiographic signs of RV overload include an augmented right-left ventricular basal diameter ratio, flattening of the interventricular septum toward the LV during systole and/or diastole, and an increased LV eccentricity index (1, 9, 10).

On the verge of a comprehensive assessment of underlying causes and unspecific symptoms related to PAH, gated myocardial SPECT may be a not-so-uncommon examination to study the LV. Nevertheless, the relatively smaller size and lower vascularization grade of the healthy RV wall in comparison to the LV myocardium renders it invisible on myocardial perfusion SPECT images. Consequently, routine imaging analysis and interpretation do not typically include the RV. In certain cases, an enhanced tracer uptake of the RV can be observed and may be an indicator of LV global perfusion deficits or RV hypertrophy, due to increased RV volumes and/or pressures (11, 12). Of note, as previously mentioned, this condition leads to altered morphologic and kinetic properties of the interventricular septum, often displaced and flattened towards the LV, and gives rise to the characteristic D-shaped LV signifying RV overload—in particular, it indicates an isolated RV volume overload if observed during systole and combined RV pressure and volume overload during diastole (6, 7).

The displacement and loss of curvature of the septum can be best assessed in short-axis parasternal views in echocardiography and analogous to the short-axis views in myocardial SPECT (7) (Figure 4). When using the latter as a diagnostic modality, this sign must be carefully distinguished to avoid misinterpreting possible septal perfusion defects or global LV perfusion decrease caused by the hypervascularization or overload of the RV (13). In our case, the small septal ischemia after stress could potentially be attributed to increased intermural pressures rather than a mere coronary insufficiency. On the other hand, this finding may shed light on RV alterations due to ischemic left heart failure, particularly in the absence of structural right heart abnormalities or pulmonary disease (8). Indeed, Williams et al. previously showed that enhanced RV tracer uptake on stress SPECT perfusion imaging in patients with CAD could indicate an exercise-induced imbalance between RV and LV perfusion, potentially associated with severe CAD (13). In such a scenario, a coronary CT could be the optimal choice to exclude CAD, while a coronary angiography would be excessive due to the weaker association between the atypical ischemic pattern observed on myocardial perfusion SPECT and epicardial CAD, as well as the limited therapeutic implications resulting from the small risk area. Considering the patient's comorbidities and normal systolic function and wall motion, except for the septum, it is most likely that other factors were contributing to the dyspnea progression. Nevertheless, without a coronary CT, the presence of CAD cannot be definitively excluded.

## Conclusion

This case presents a unique scenario in routine myocardial SPECT imaging and warns us that the pathologies in the RV might impede the evaluation of the LV. The two ventricles are interconnected and the RV—although not frequently observed in SPECT perfusion images—influences morphology, motion, and perfusion of the LV in gated myocardial SPECT. The Movahed's sign or the D-shaped LV due to the flattening of the interventricular septum indicates the intricate and dynamic nature of the heart function.

This case represents the first approach to the simultaneous evaluation of symptom origin within the context of cardiovascular risk factors, and PH under vasodilation therapy (Treprostinil) using routine cardiac perfusion imaging. Furthermore, it emphasizes the need for nuclear medicine physicians to consistently consider and integrate the Movahed's sign and other RV-related anomalies into the correct interpretation and reporting of images, recognizing the right heart changes in pathologies such as PAH and the diagnostic value of SPECT imaging.

## Data Availability

The original contributions presented in the study are included in the article/Supplementary Material, further inquiries can be directed to the corresponding author.
